# Zinc is a critical regulator of placental morphogenesis and maternal hemodynamics during pregnancy in mice

**DOI:** 10.1038/s41598-017-15085-2

**Published:** 2017-11-09

**Authors:** Rebecca L. Wilson, Shalem Y. Leemaqz, Zona Goh, Dale McAninch, Tanja Jankovic-Karasoulos, Gabriela E. Leghi, Jessica A. Phillips, Katrina Mirabito Colafella, Cuong Tran, Sean O’Leary, Sam Buckberry, Stephen Pederson, Sarah A. Robertson, Tina Bianco-Miotto, Claire T. Roberts

**Affiliations:** 1 The Robinson Research Institute, University of Adelaide, Norwich Centre, 55 King William St, North Adelaide, South Australia 5006 Australia; 2 Adelaide Medical School, Department of Obstetrics and Gynaecology, University of Adelaide, Adelaide, South Australia 5005 Australia; 3 Waite Research Institute and School of Agriculture, Food and Wine, University of Adelaide, Waite Campus, Urrbrae, South Australia 5064 Australia; 4 Cardiovascular Program, Monash Biomedicine Discovery Institute, Monash University, Melbourne, Victoria 3800 Australia; 5 Department of Physiology, Monash University, Melbourne, Victoria 3800 Australia; 6 CSIRO Health and Biosecurity, Gate 13 Kintore Ave, Adelaide, 5000 Australia; 7 Adelaide Medical School, Discipline of Physiology, University of Adelaide, Adelaide, South Australia 5005 Australia; 8 Harry Perkins Institute of Medical Research, University of Western Australia, Crawley, Western Australia 6009 Australia; 9 Australian Research Council Centre of Excellence in Plant Energy Biology, University of Western Australia, Western Australia, 6009 Australia; 10 Bioinformatics Hub, School of Biological Sciences, University of Adelaide, Adelaide, South Australia 5005 Australia

## Abstract

Zinc is an essential micronutrient in pregnancy and zinc deficiency impairs fetal growth. We used a mouse model of moderate zinc deficiency to investigate the physiological mechanisms by which zinc is important to placental morphogenesis and the maternal blood pressure changes during pregnancy. A 26% reduction in circulating zinc (P = 0.005) was exhibited in mice fed a moderately zinc-deficient diet. Zinc deficiency in pregnancy resulted in an 8% reduction in both near term fetal and placental weights (both P < 0.0001) indicative of disrupted placental development and function. Detailed morphological analysis confirmed changes to the placental labyrinth microstructure. Continuous monitoring of maternal mean arterial pressure (MAP) revealed a late gestation decrease in the zinc-deficient dams. Differential expression of a number of regulatory genes within maternal kidneys supported observations on MAP changes in gestation. Increased MAP late in gestation is required to maintain perfusion of multiple placentas within rodent pregnancies. Decreased MAP within the zinc-deficient dams implies reduced blood flow and nutrient delivery to the placenta. These findings show that adequate zinc status is required for correct placental morphogenesis and appropriate maternal blood pressure adaptations to pregnancy. We conclude that insufficient maternal zinc intake from before and during pregnancy is likely to impact *in utero* programming of offspring growth and development largely through effects to the placenta and maternal cardiovascular system.

## Introduction

During pregnancy, the placental vasculature provides the interface between the fetus and the mother for exchange of nutrients and wastes. Adequate placental function underpins normal fetal development^1^. Defects in placental development and function are implicated in a number of clinical pregnancy complications. These include preeclampsia (PE)^2^, a common hypertensive disorder of pregnancy^3^ and fetal growth restriction (FGR)^4–7^, defined as birth weight, adjusted for gestational age, of ≤5^th^ percentile^3^. Together these conditions pose a lifelong risk of morbidity and mortality for both the mother and infant. Increased placental oxidative stress is hypothesised to be an underlying cause of pathogenesis in pregnancies complicated by PE and FGR^8^. While the precise mechanisms remain unclear, it is highly likely that micronutrient deficiencies play a pivotal role.

Zinc is extremely important during the accelerated fetal growth phase characteristic of late gestation. It is an essential element in more than 1000 proteins required for biological functions including antioxidant defence, cell signalling and gene expression^9–12^. In development, zinc is not only important for the action of transcription factors and the enzymes which catalyse the synthesis of DNA and RNA, but also as a component of the accessory proteins that regulate the activation of key development genes^13^. Increased fetal loss and high rates of congenital malformations in several organs of surviving fetuses are characteristics of severe zinc deficiency in pregnancy^14,15^. Compromised DNA integrity and increased oxidative stress are likely drivers of impaired tissue and physiological function when zinc intake is insufficient, since zinc is an essential component of copper-zinc superoxide dismutase (Cu/Zn-SOD)^16^, and DNA-repair mechanisms such as p53^17,18^.

In rodents, maternal zinc deficiency consistently causes reduced fetal growth evident from mid-gestation to near term^19–23^. The mechanisms by which placental development and function contributes to impaired fetal growth in these models is largely unknown. Maternal hemodynamic adaptations to pregnancy are essential for optimal placental development and function, since adequate blood flow to the placenta underpins normal fetal growth^24^. Reduced uterine blood flow to the placenta not only constrains delivery of nutrients to the fetus and increases fetal hypoxia but also has implications for production of factors which modulate placental vascular growth^25^. We hypothesised that reduced fetal growth associated with maternal zinc deficiency is modulated by altered placental development and function and aimed to characterise the effect of moderate maternal zinc deficiency on placental morphogenesis and maternal cardiovascular adaptations to pregnancy, in particular maternal blood pressure.

## Results

### Moderate Dietary Zinc Restriction Reduces Circulating Zinc and Liver Metallothionein Concentrations

**Figure 1:**
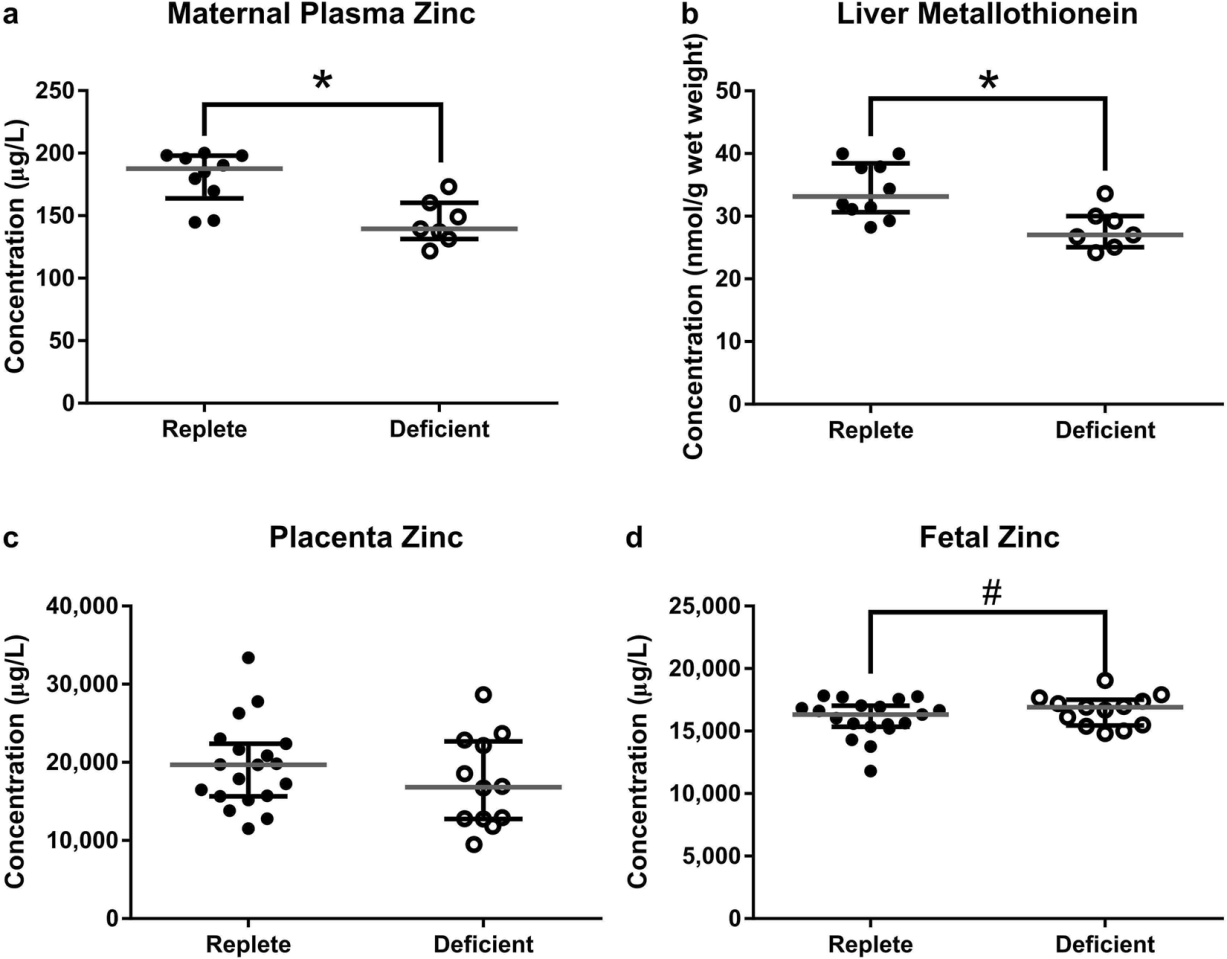
Lower zinc in the diets of the zinc-deficient mice reduced both short-term and long-term zinc stores. At GD18.5, a reduction in circulating zinc levels (**a**) as well as liver metallothionein (**b**) was observed in the zinc-deficient dams. Placental tissue zinc however remained similar between the two diet groups (**c**) and there was a trend for an increase in zinc content of fetuses from zinc-deficient dams (**d**). Data are median and interquartile range (n = 10 zinc-replete and 7 zinc-deficient [a & b] and n = 19 zinc-replete and 13 zinc-deficient [c & d]). Statistical significance was determined using Mann-Whitney Test on data based on an average litter size of 7.00. *P < 0.05, ^#^ *P* = 0.08.

To confirm that moderate dietary zinc restriction reduced maternal zinc status in C57BL/6 J female mice fed a zinc-deficient diet (containing 10 mg/kg zinc) compared to a zinc-replete diet (containing 40 mg/kg zinc), we analysed maternal plasma zinc concentrations, liver metallothionein expression and placental and fetal zinc content. At gestational day (GD) 18.5, there was a 26% decrease in circulating zinc in the zinc-deficient dams when compared to the zinc-replete dams (Fig. 1a; *P* = 0.005) and long term zinc status, as measured by liver metallothionein, was also reduced by 19% (Fig. 1b; *P* < 0.0001). There was no difference in maternal food consumption across the experimental period (mean 7 day food consumption per cage containing 4 mice (SEM) deficient: 92.4 (1.29) vs. replete: 90.6 (1.86) g; *P = *0.45), confirming reduced zinc status was not a consequence of anorexia. Placental zinc concentrations remained similar between the two diet groups (Fig. 1c; *P* = 0.59) and there was a trend for an increase in zinc concentrations in fetuses from the zinc-deficient dams (Fig. 1d; *P* = 0.08), suggesting active mechanisms for sequestering zinc into gestational tissues. Circulating levels of phosphorus, sulphur and potassium were slightly higher in the zinc-deficient dams (Supplementary Table S1) but was likely due to minor differences in the diet compositions (Supplementary Table S2) and these nutrients did not differ in placental or fetal tissues (Supplementary Table S1).

### Moderate Maternal Zinc Deficiency Impairs Fetal Growth

**Figure 2:**
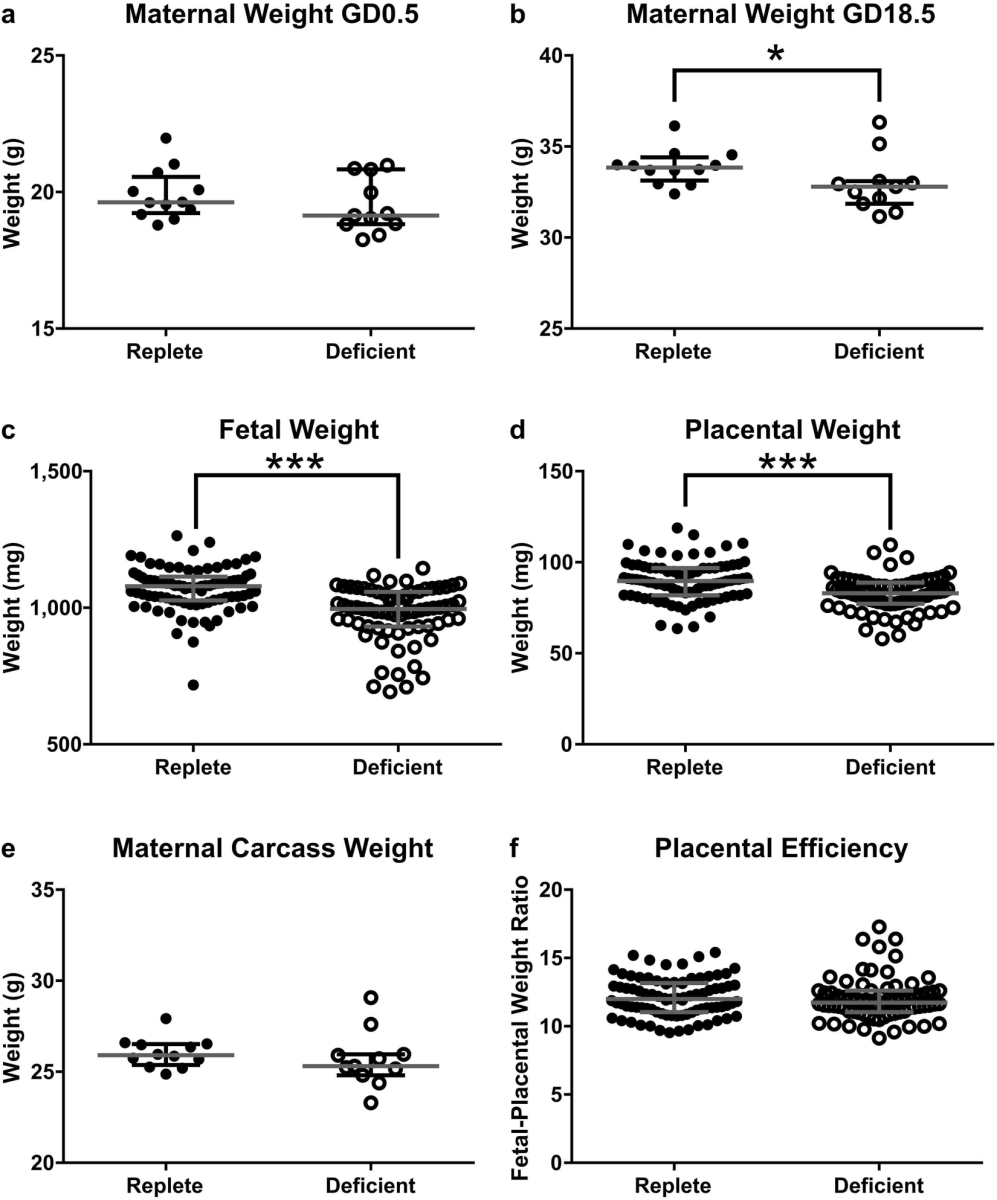
Maternal zinc deficiency altered reproductive outcome, measured at GD18.5. No significant difference in maternal weight was observed between the zinc-replete and zinc-deficient dams at mating (**a**). At GD18.5, maternal weight of the zinc-deficient dams was reduced compared to the replete (**b**). This was largely due to a decrease in both fetal (**c**) and placental (**d**) weight as maternal carcass weight (**e**) was not significantly different. Placental efficiency, measured by the fetal-placental weight ratio was not different between the two diet groups (**f**). Data are median and interquartile range (n = 12 zinc-replete and 11 zinc-deficient). Statistical significance was determined using Mann-Whitney Test on data based on an average litter size of 7.00. *P < 0.05, ***P < 0.001.

To investigate the impact of zinc deficiency during pregnancy on placental development, we first compared reproductive outcomes in zinc-deficient and zinc-replete dams. At GD0.5 the zinc-deficient dams did not differ in weight compared to the zinc-replete dams (Fig. 2a; *P* = 0.24). However, at GD18.5 (24 hours before birth) they were 3% lighter compared to the zinc-replete (Fig. 2b; *P* = 0.044). The proportion of mated mice exhibiting viable pregnancies in late gestation was not different (data not shown), and litter size was not significantly different between the two diet groups (median [IQR] zinc-deficient: 7 [5, 8] vs. zinc-replete: 8 [6, 8.75]; *P* = 0.36), indicating no impact on fertility or fecundity. The lighter maternal body weight in the zinc-deficient dams was largely accounted for by an 8% reduction in both fetal and placental weights (Fig. 2c,d; both *P* < 0.0001) as maternal carcass weight (maternal weight minus fetal and placental weights combined) did not differ between the two diet groups (Fig. 2e; *P* = 0.24). The fetal-placental weight ratio did not significantly differ (Fig. 2f; *P* = 0.21), indicating similar nutrient transport efficiencies between the placentas. The importance of zinc to growth and development was highlighted in the postnatal phase, with pups from the zinc-deficient mothers 32% lighter at weaning compared to pups born to control dams (Supplementary Fig. S1). Survival rates to weaning were similar regardless of zinc status (survival at weaning; zinc-deficient: 30 out of 40 (75%) vs. zinc-replete: 20 out of 27 (74%); χ^2^ analysis; *P* = 1).

### Effect of Maternal Zinc Deficiency on Placental Morphology

**Figure 3:**
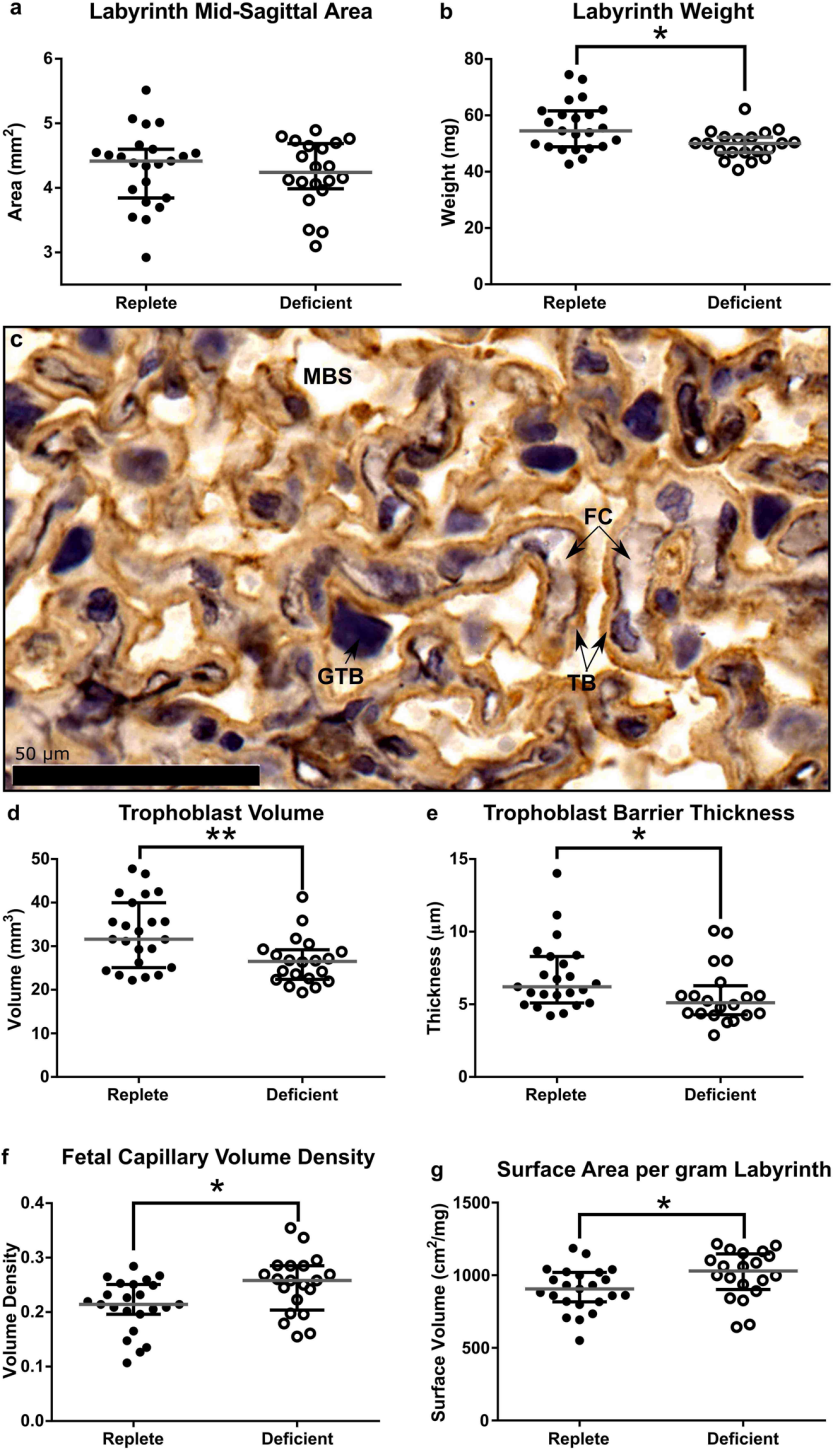
Maternal zinc deficiency during pregnancy resulted in changes to the placental architecture likely to affect fetal growth. Despite no significant different in the labyrinth zone mid-sagittal cross sectional area (**a**), labyrinth zone weight was reduced in the zinc-deficient placentas at GD18.5 (**b**). Double labelling immunohistochemistry was used to identify the fetal capillaries (FC), trophoblasts (TB) and maternal blood space (MBS) within the labyrinth zone (**c**). Analysis revealed decreases in the trophoblast volume (**d**) as well as trophoblast barrier thickness (**e**) in the placentas from zinc-deficient dams. An increase in fetal capillary volume density (**f**) and surface volume (**g**) was also found. Data are median and interquartile range (n = 23 and 21 placentas from 12 zinc-replete and 11 zinc-deficient dams, respectively). Statistical significance was determined using Mann-Whitney Test on data based on an average litter size of 7.00. *P < 0.05, **P < 0.01. GTB: giant trophoblast cells.

Having confirmed that moderate zinc deficiency in pregnancy resulted in reduced placental weight, we analysed placental morphology at GD18.5 as FGR is often associated with poor placental morphogenesis, and underpins future placental function^26^. Total placental mid-sagittal cross sectional area was not significantly different between the two diet groups despite a reduction in placental weight (median [IQR], deficient: 7.17 [5.88, 7.35] vs. replete: 6.71 [6.18, 7.01] mm^2^; *P* = 0.21). Furthermore, there was no difference in the mid-sagittal cross sectional area of the labyrinth zone; the region responsible for nutrient and waste exchange (Fig. 3a; *P* = 0.69), despite an 8% reduction in labyrinth zone weight in placentas from zinc-deficient dams (Fig. 3b, *P* = 0.012). This implied a disproportionate shift in placental architecture, as commonly occurs in an adaptive response to nutritional perturbation^27^, and was supported by detailed immunohistochemical (IHC) analysis. There are three main compartments which comprise the labyrinth zone; the fetal capillaries (FC: fetal circulation), maternal blood space (MBS: maternal circulation) and the trophoblasts which act as a barrier between the FC and MBS, to coordinate nutrient and waste exchange (Fig. 3c). In the placentas of zinc-deficient dams, both trophoblast volume and trophoblast barrier thickness were reduced by 16% (Fig. 3d,e; *P* = 0.007 and *P* = 0.016, respectively) there was a 17% increase in the volume density of FC (Fig. 3f; *P* = 0.02) and 10% increase in surface area density (Fig. 3g; *P* = 0.028) compared to placentas from zinc-replete dams. Together these results indicate a compensatory mechanism in the zinc-deficient placentas that nevertheless was unable to rescue fetal growth.

### Placental Iron Transport is Reduced by Zinc Deficiency

**Figure 4:**
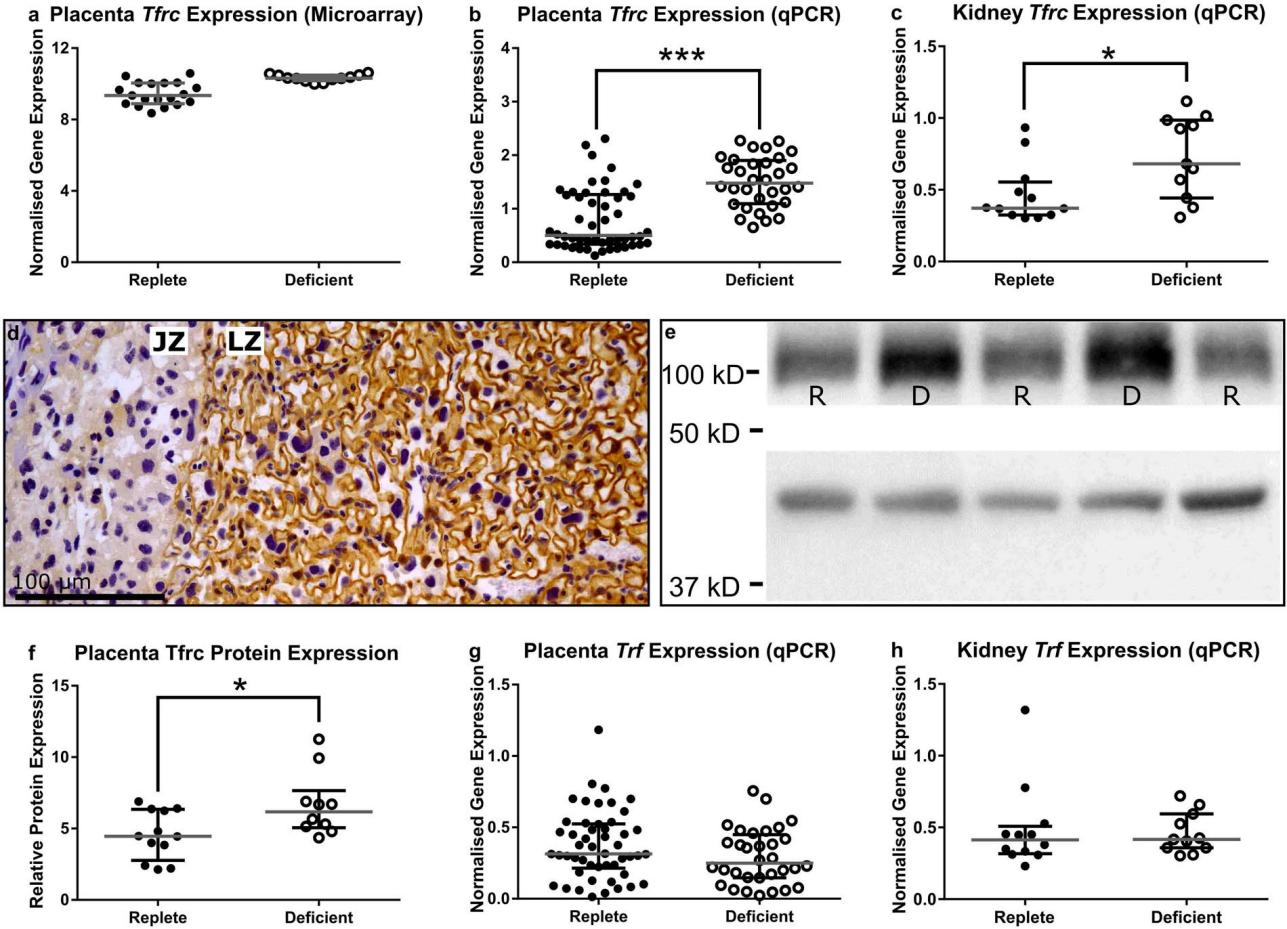
Microarray analysis revealed an 8.5-fold increase in gene expression of transferrin receptor (*Tfrc*) in the placentas of the zinc-deficient dams (**a**). This increase was validated and confirmed in an independent cohort of placental samples using qPCR (**b**). qPCR was also used to measure *Tfrc* expression within kidney tissue at GD18.5 and was also increased in tissue collected from zinc-deficient dams (**c**). Immunohistochemical analysis of Tfrc protein showed localisation to the apical surface of the trophoblast cells within the labyrinth zone (LZ) of the placenta (**d**). Tfrc protein expression was analysed using Western blot and compared to β-actin expression (**e**); Tfrc band at ~100 kD and β-actin at ~42 kD, (full image in supplementary information). This revealed an increased expression of Tfrc protein in placentas from zinc-deficient dams (**f**). Transferrin (*Trf*) gene expression within the placenta and kidney was also quantified by qPCR but did not differ between the two diet groups (**g**,**h**). Data are median and interquartile range (n = 19 and 12 placentas [a]; 50 and 32 placentas [b and g]; 12 and 11 kidneys [c and h] and 12 and 10 placentas [e and f] from 12 zinc-replete and 11 zinc-deficient dams, respectively). Statistical significance was determined using Mann-Whitney Test. *P < 0.05, ***P < 0.001. JZ: junctional zone.

Zinc is extremely important in regulating the expression of a number of development genes crucial for fetal growth^13^. Conservation of zinc within the placentas and fetuses of the zinc-deficient dams suggested reduced zinc in the gestational tissues was not driving the reduction in fetal growth. A microarray analysis was used to identify pathways within the placenta that may be disrupted by maternal zinc deficiency however, only eight genes were identified to be potentially differentially expressed (Supplementary Table S3). An 8.5-fold increase in the expression of *Transferrin Receptor* (*Tfrc*) in placentas from zinc-deficient dams was identified (Fig. 4a and Supplementary Table S3) and qPCR validation in an independent cohort of placentas confirmed an increase in gene expression (Fig. 4b; *P* < 0.0001). Furthermore, there was a 36% increase in the expression of *Tfrc* in maternal kidney tissue from the same cohort of mice (Fig. 4c; *P* = 0.019). IHC staining showed Tfrc protein localised to the apical surface of the trophoblast cells within the placental labyrinth (Fig. 4d) and Western blot analysis confirmed a 32% increase in protein expression in zinc-deficient placentas compared to zinc-replete (Fig. 4e and f; *P* = 0.022). Increased *Tfrc* expression was most likely a response to decreased placental and fetal iron concentration in the tissues from zinc-deficient dams (Supplementary Table S1). A 24% (*P* = 0.048) and 20% (*P* = 0.001) decrease in the zinc-deficient dams in placental and fetal iron, respectively, indicated disruption to placental iron transport despite maternal circulating iron remaining similar between the two diet groups (Table S1; *P* = 0.42). These data suggest a possible perturbation in the expression of Transferrin (Trf) protein which binds Tfrc to move predominantly iron into cells. However, using qPCR, we did not observe differential expression of *Trf* in either placentas or kidneys between the two diet groups (Fig. 4g and h; *P* = 0.12 and *P* = 0.82, respectively).

### DNA Damage Caused by Oxidative Stress is Elevated by Zinc Deficiency

**Figure 5:**
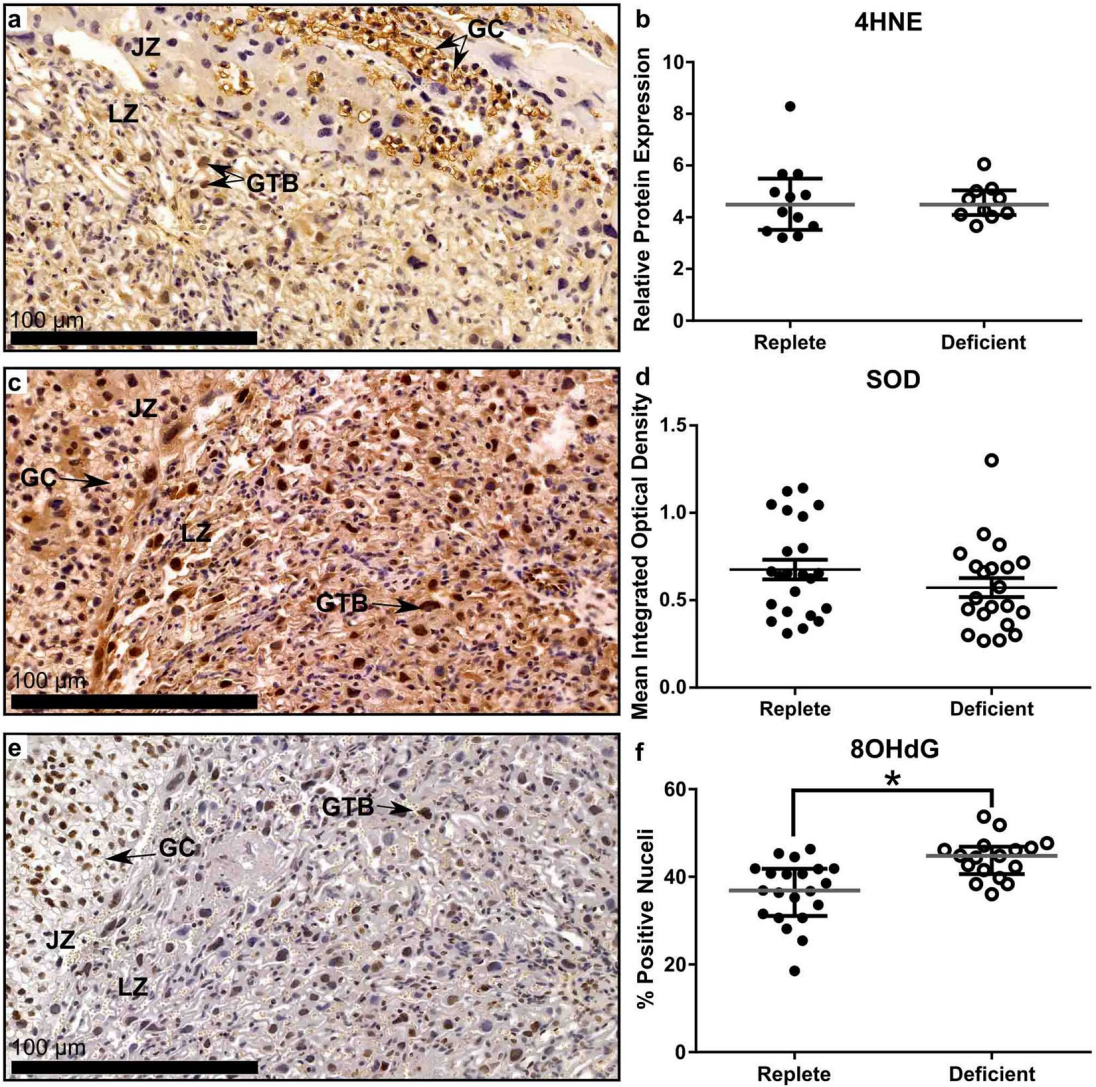
Maternal zinc deficiency resulted in increased DNA damage caused by oxidative stress within the placenta at GD18.5. 4-hydoxynoneal (4HNE) expression was localisation to the glycogen cells (GC) present in the junctional zone (JZ) and giant trophoblast cells (GTB) within the labyrinth zone (LZ) of the placenta (**a**). Western blot analysis showed no difference in 4HNE expression between the two diet groups (**b**). Expression of Cu/Zn-Superoxide Dismutase (SOD) was found throughout the placenta (**c**) but expression was not different between placentas collected from a zinc-deficient or zinc-replete dams (**d**). 8-hydroxy-deoxyguanosine (8OHdG) was also expressed by the GTB cells and GC within the junctional zone (**e**). There was a statistically significant increase in the percentage of positive cells in the labyrinth zone of the placentas from zinc-deficient dams indicating an increase in DNA damage caused by oxidative stress (**f**). Data are median and interquartile range (n = 12 and 10 placentas [b] and 23 and 21 placentas [d & f] from 12 zinc-replete and 11 zinc-deficient dams, respectively). Statistical significance was determined using Mann-Whitney Test. *P < 0.05.

*In vitro* and *in vivo* models of both zinc and iron deficiency reveal increased oxidative stress and DNA damage^28–32^ as well as increased oxidative stress within the placenta in pregnancies complicated by FGR^33,34^. We investigated whether disrupted placental function in this model of zinc deficiency was associated with increased oxidative stress. Placental expression of 4-hydroxynonenal (4HNE; a product of lipid peroxidation and marker of oxidative stress) was largely localized to the giant trophoblasts within the labyrinth zone (Fig. 5a) however, Western blot quantification of 4HNE expression revealed no difference in relative protein expression between placentas from zinc-deficient and zinc-replete dams (Fig. 5b; *P* = 0.72). There was also no difference in the expression of Cu/Zn-SOD between placentas from the two diet groups (Fig. 5c,d; *P* = 0.28). This indicates that neither increased lipid peroxidation nor reduced SOD expression was a characteristic of placental dysfunction in this model. However, increased DNA damage within the placental labyrinth zone in the placentas from the zinc-deficient dams as indicated by increased nuclei stained positively for 8-hydroxy-2′-deoxyguanosine (8-OHdG) (Fig. 5e,f; *P* < 0.0001) suggests a reduction in cellular integrity mediated by increased oxidative stress which would contribute to placental dysfunction. 8-OHdG staining was localised predominantly within giant trophoblasts as well as within glycogen cells present in the junctional zone.

### Maternal Zinc Deficiency Alters Maternal Blood Pressure Profile During Pregnancy and Lactation

**Figure 6:**
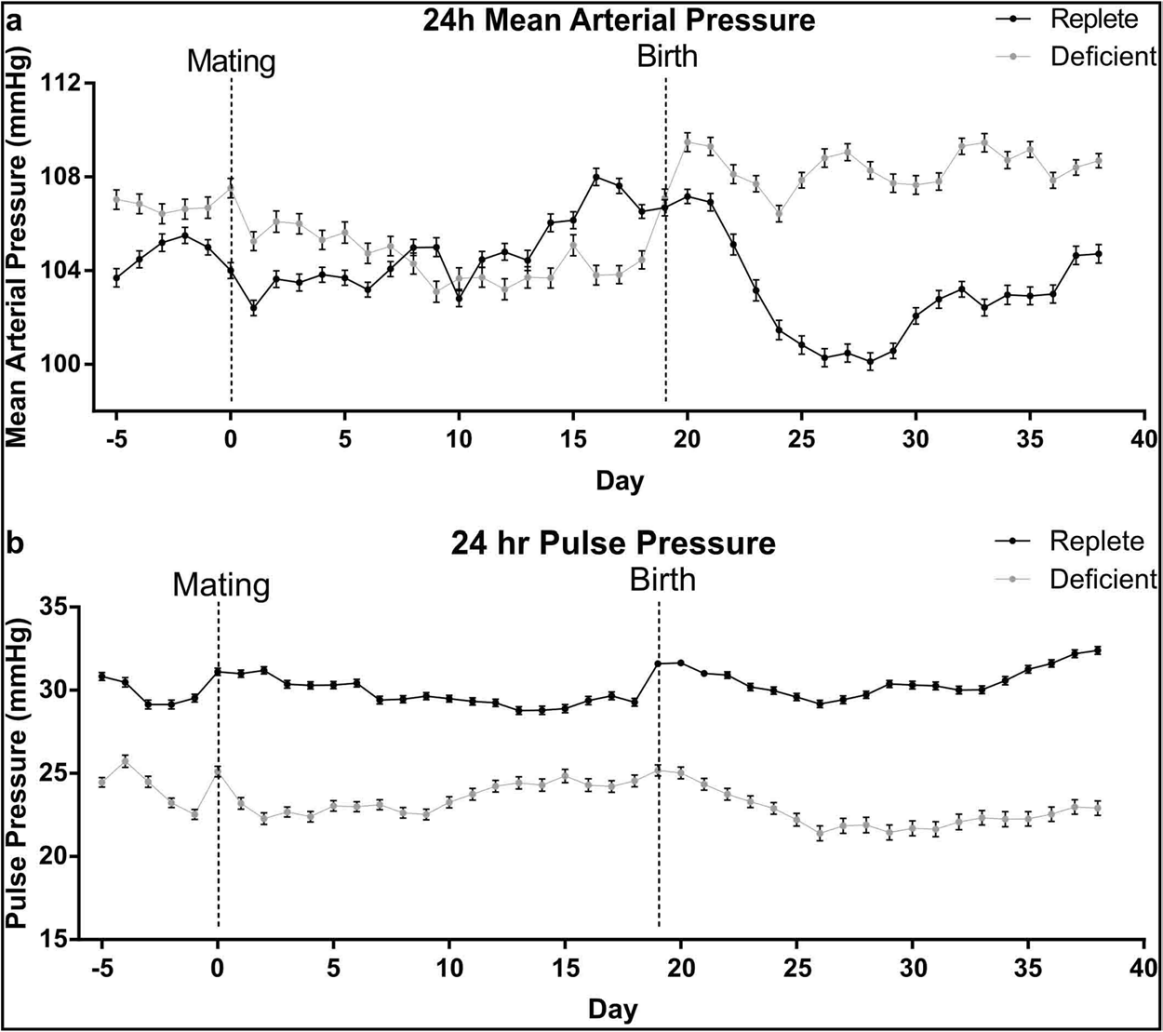
Maternal zinc deficiency changed the blood and pulse pressure profiles of dams before pregnancy, during pregnancy and in lactation. Prior to pregnancy and during lactation, 24 hour mean arterial pressure (MAP) was significantly elevated in the zinc-deficient dams. (**a**) During pregnancy, from days GD0 to GD5, MAP was significantly elevated in the zinc-deficient dams. However, across GD6 to GD10, MAP began to decrease in the zinc-deficient dams while an increase was observed in the zinc-replete dams. This resulted in significantly lower MAP in the zinc-deficient dams between GD11 to GD19 of pregnancy. (**b**) Pulse pressure, which is the difference between systolic and diastolic blood pressures, was significantly lower in the zinc deficient animals across the whole experimental period. Each data point represents the average 24 h MAP or pulse pressure for each diet group ± SEM. n = 4 zinc-replete and 4 zinc-deficient dams. Statistical differences were determined using a general additive model.

Adequate blood flow to the placenta is crucial for the maintenance of fetal growth and an exponential increase in placental blood flow is necessary for transport of oxygen, water and nutrients in late gestation^25^. Maternal cardiovascular adaptations to pregnancy are crucial in maintaining appropriate placental blood flow so we sought to investigate whether zinc deficiency in pregnancy altered maternal blood pressure. Continuous measurements of mean arterial pressure (MAP), systolic blood pressure (SBP) and diastolic blood pressure (DBP) prior to pregnancy, within pregnancy and across lactation revealed that blood pressure was disturbed without adequate zinc intake (Fig. 6a and Supplementary Figure S2a). Across the five days prior to mating, average 24 h MAP was elevated in the zinc-deficient dams compared to zinc-replete dams (Fig. 6a), driven by increased DBP during this period (Supplementary Fig. S2a). 24 h MAP, SPB and DBP were also elevated in the zinc-deficient dams during lactation. Within pregnancy, regardless of diet, MAP was different between days 11–19 of pregnancy compared to days 1–5. Between days 6–10 of pregnancy, 24 h MAP was elevated in the zinc-replete dams but decreased in deficient dams. This resulted in lower 24 h MAP between days 11–19 of pregnancy in the zinc-deficient dams compared to zinc-replete dams (Fig. 6a). Further analysis of diurnal MAP pattern during the day (0700 h–1850 h) and night (1900 h–0650 h) revealed differences in MAP during pregnancy were driven by differences in MAP during the day when mice are at rest and not during the night when mice are active (Supplementary Fig. S2b). Average 24 h pulse pressure; the difference between SBP and DBP, was lower in the zinc-deficient dams across the whole experimental period when compared to the zinc-replete dams (Fig. 6b). This, along with the fact that 24 h heart rate (HR) was also decreased in the zinc-deficient dams (Supplementary Fig. S2c) indicates a potential reduction in cardiac output and reduced organ perfusion.

### Maternal Zinc Deficiency Affects Renal Gene Expression and Structure

**Figure 7:**
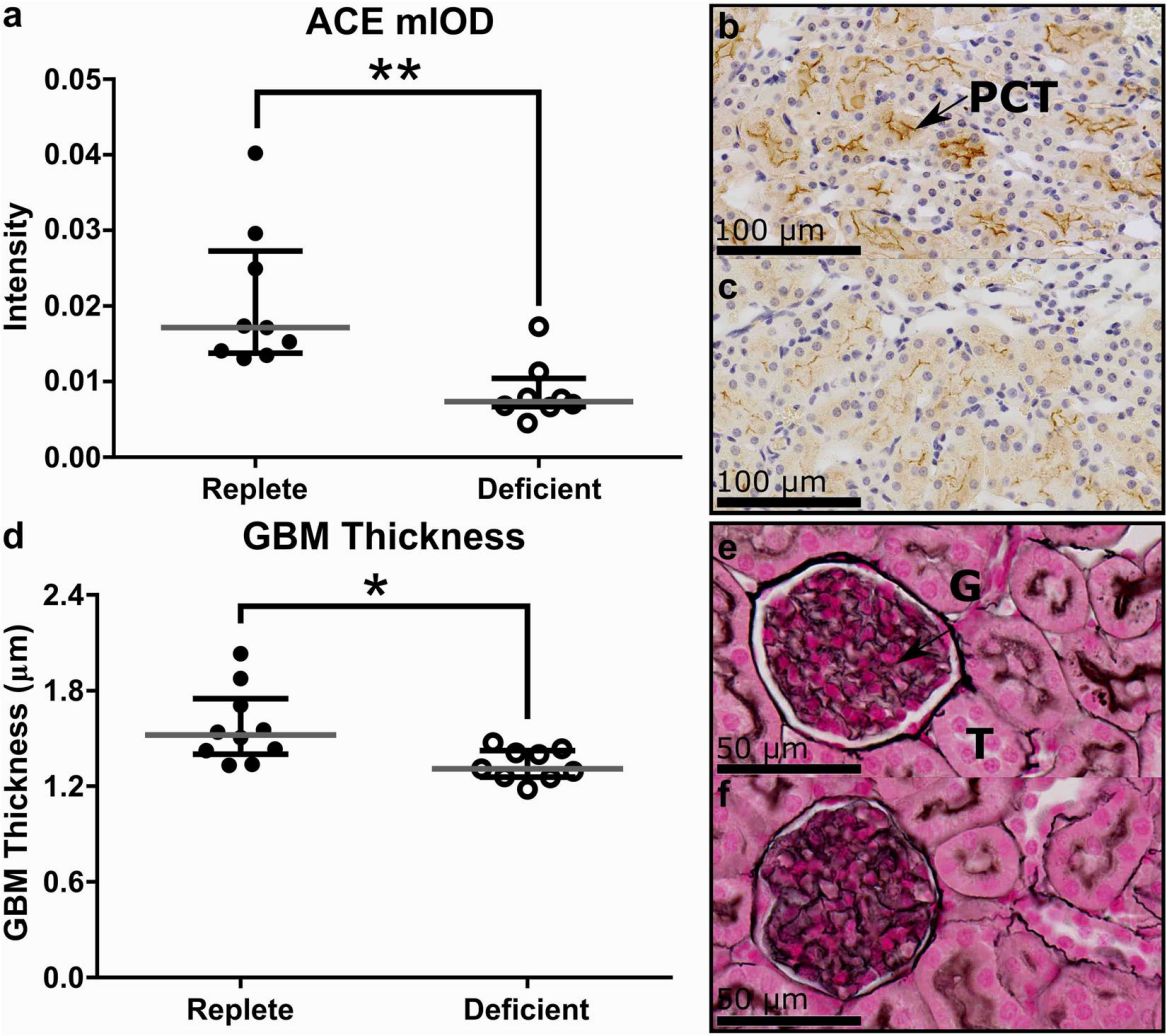
Effects of marginal zinc deficiency on renal parameters at GD18.5. Decreased gene expression of *angiotensin converting enzyme* (*Ace*) was associated with a decrease in Ace protein expression within the kidneys of zinc-deficient dams (**a**). Representative image of Ace expression in a zinc-replete (**b**) and zinc-deficient (**c**) kidney section. Ace protein was localised to the proximal convoluted tubules (PCT). Marginal maternal zinc deficiency also decreased glomeruli basement membrane thickness (**d**). Representative images of a glomerulus in a zinc-replete (**e**) and zinc-deficient (**f**) kidney section. Data are median and interquartile range (n = 10 zinc-replete and 9 zinc-deficient dams). Statistical significance was determined using Mann-Whitney Test. *P < 0.05, **P < 0.01. G: glomerulus. T: tubule

To investigate whether changes in maternal blood pressure late in gestation may be influenced by effects on aspects relating to renal function, we analysed kidney gene expression and morphology. qPCR analysis of kidney tissue at GD18.5 identified a number of differentially expressed genes in zinc-deficient and zinc-replete dams associated with blood pressure regulation (Supplementary Table S4). In particular, a 16% reduction in kidney expression of *angiotensin converting enzyme* (*Ace*) (*P* = 0.031) and a 44% reduction in *inositol 1,4,5-triphosphate receptor type 2* (*Itpr2*) (*P* = 0.05) in the zinc-deficient dams occurred in association with reduced blood pressure observed at GD18.5 in mice implanted with blood pressure monitors. Ace protein expression in the kidneys was localised to the apical brush-borders of the proximal convoluted tubules and a 57% reduction in Ace protein expression in the kidneys of the zinc-deficient dams was demonstrated when compared to the zinc-replete dams (Fig. 7a–c; *P* < 0.01). Zinc deficiency also caused changes to glomerular morphology which can have implications for blood pressure. An 11% reduction in glomeruli basement membrane (GBM) thickness was observed in the zinc-deficient dams, compared to the zinc-replete dams at GD18.5 (Fig. 7d–f; *P* < 0.01), which would affect glomerular filtration capabilities and therefore maternal blood pressure. Overall, this data is consistent with blood pressure measurements in late gestation. Given that placental blood flow is dependent upon maternal hemodynamic alterations across gestation, the effects of zinc deficiency on maternal blood pressure are likely to contribute to placental dysfunction.

### Maternal Zinc Deficiency Alters Immunological Parameters in Late Gestation

In addition to renal function, maternal blood pressure in pregnancy is influenced by immune cells, particularly T cells and natural killer (NK) cells^35^. These cells impact maternal hemodynamics and placental development by suppressing inflammatory mediators in the implantation site^36^ and promoting appropriate uterine vascular adaptation^37^. Spleen hypotrophy, as indicated by a 50% decrease in maternal spleen weight as a percentage of carcass weight, was observed in the zinc-deficient dams compared to zinc-replete dams at GD18.5 (Supplementary Fig. S3a; *P = *0.01). There was also a significant reduction in liver, kidney and lung weights as a percentage of carcass weight in the zinc-deficient dams suggesting aberrant maternal adaptation to pregnancy (Supplementary Fig. S3b-d; *P* < 0.05 for all). The failure to exhibit the spleen hypertrophy typical of late gestation^38,39^ is consistent with an impaired immune response to pregnancy. Histological analysis of the spleen at GD18.5 showed no difference in the percentage area of white pulp, the splenic region populated by lymphocytes, between the two diet groups (Supplementary Fig. S4a). However, there was a decrease in the absolute number of cells, calculated based on splenic weight, within the white pulp in the spleens of the zinc-deficient animals compared to the zinc-replete indicative of a reduced lymphocyte pool (Supplementary Fig. S4b; *P* = 0.051). We investigated changes to immune cell populations within the spleen at GD18.5 using qPCR to analyse marker genes and found a 41% reduction in the expression of *Fasl*, a marker expressed by activated T cells and NK cells and a 1.26-fold increase in the expression of *Il10*, which is expressed by regulatory T cells and type 2 T helper cells, in the zinc-deficient dams (Supplementary Fig. S5a,b; *P* = 0.043 for both, respectively). Furthermore, a trend toward elevated *Ifna1* (Supplementary Fig. S5c; *P* = 0.07) expression was observed in the spleens of zinc-deficient dams compared to controls. This data indicates a possible imbalance in the residual lymphocyte subpopulations^40^ which would be expected to contribute to the cardiovascular and placental anomalies seen in the zinc-deficient dams. Maternal immune adaptations to pregnancy are crucial to placental development during the implantation period and an altered immune balance may contribute to the mechanisms through which zinc deficiency disturbs placental morphogenesis and fetal growth.

## Discussion

Correct placental morphogenesis underpins pregnancy success and offspring phenotype through tight regulation of transport of nutrients, gases and waste between the mother and fetus. This study confirms that maternal zinc status is a key determinant of fetal growth and shows that the effects of limited zinc are likely to be mediated through adverse placental development and function. Additionally, we show that altered placental development is associated with, and likely to be a consequence of, combined effects of altered maternal cardiovascular adaptations to pregnancy and altered renal function and immune changes. An interaction between zinc and other micronutrients within the placenta has also been identified, demonstrating the existence of micronutrient bioavailability interactions within this complex physiological network.

Reduced fetal growth has been described in a number of rodent models assessing the effect of marginal and severe dietary zinc restriction during pregnancy^19–23^. In our model, reduced fetal growth at GD18.5 was associated with an 8% reduction in placental weight, a common phenotype of growth restriction in humans and animals^26,41^. We sought to further investigate perturbations in placental development by defining impact on the placental architecture which regulates nutrient transport and fetal growth. A lower placental weight was reported at GD12.5 in a model of severe zinc restriction (<1 mg/kg)^21^. Unlike this earlier study, we did not observe a difference in labyrinth zone area at GD18.5 but IHC analysis of labyrinth zone composition demonstrated a change in placental structure indicative of a compensatory mechanism. Nutrients transfer from the maternal circulation to the fetus requires passage through a layer of trophoblast cells which surround the fetal capillaries^42^. Increases to the exchange surface area and the volume density of fetal capillaries, as well as decreases in the trophoblast barrier thickness of the zinc-deficient placentas suggest an increase in the transfer efficiency of nutrients per gram of placenta. However, the reduction in total placental size and thus labyrinthine volume is likely to elicit a greater effect on the transport of nutrients to the fetus than the difference in the magnitude of nutrient transfer per gram of placenta. This is supported by the observed similar placental efficiency between dietary zinc groups, as measured by the fetal-placental weight ratio, indicating these compensatory changes to the labyrinth zone structure are insufficient to rescue fetal growth.

The structure of the placenta at term is determined by events that occur much earlier in placental morphogenesis to drive differentiation of various trophoblast cell lineages and mesenchymal cells from stem cells in a defined stochastic relationship^26,43^. Alterations in gross placental structure as well as to the ultrastructure of the labyrinthine zone can confer either a functional advantage or disadvantage for nutrient transfer and fetal growth. Changes in developmental patterning that result in altered surface area, barrier thickness and fetal capillary volume can in part be influenced by the maternal immune adaptation to pregnancy, and the capacity of immune cells to support or limit early placental development^44^. Since the spleen is a key source of NK cells and T lymphocyte recruited into the uterine decidua and gestational tissues^35^, the reduced reservoir of splenic lymphocytes and the altered gene expression in spleen cells found in zinc-deficient females suggests that the immune response is aberrant in zinc-deficient mice. This is consistent with observations of the impact of zinc on T cell responses in non-pregnant animals^45–47^. Given the critical impacts of lymphocytes on maternal vascular remodeling and systemic cardiovascular adaptations to accommodate pregnancy^35^ the effects of zinc deficiency mediated via immune cells may contribute to placental dysmorphogenesis.

In pregnancy, placental function and the maternal circulatory system are intricately dependent upon one another. Perturbations in placental function are likely to influence the maternal circulation and vice-versa^24^. Increases in blood pressure late in gestation are a characteristic of rodent pregnancies as indicated by the blood pressure profile in the zinc-replete dams of this experiment and by others^48^. This presumably facilitates maintenance of adequate perfusion of multiple placental beds as mice are litter-bearing species. Reduced uterine blood flow can also be used as a predictor of FGR in human pregnancies^49,50^ as transport of oxygen and nutrients needs to keep pace with fetal growth and this can only occur with adequate blood flow to the placenta. The decrease in blood pressure during late pregnancy, as well as reduced pulse pressure which was observed in the zinc-deficient dams is likely to result in reduced blood flow to the placenta, impairing nutrient delivery. The renin-angiotensin system (RAS) is one of the major physiological regulators of blood pressure mediated by the kidneys. Zinc is important to the function of a number of the RAS enzymes which catalyse the production of vasoconstrictors thereby increasing blood pressure^51^. Ace is a zinc metalloenzyme and chelation of zinc inhibits Ace functionality^52^. We also identified a reduction in *Itpr2* expression which is a protein that controls vascular tone and is thought to alter the responsiveness of cells to components of the renin-angiotensin system^53^. These data support the importance of zinc in correct functioning of the RAS, particularly in the kidneys, and therefore blood pressure control which is necessary to maintain adequate placental perfusion and transplacental nutrient exchange.

Not only is blood flow a crucial determinant of placental function and fetal growth, but it also has implications for oxidative stress. Fluctuations in the perfusion of the placenta and therefore oxygenation is a major inducer of oxidative stress within the placenta which can severely affect placental transport capabilities^54^ and increased oxidative stress has been characterised in the placenta of human pregnancies complicated by FGR^33,34^. Zinc itself is capable of acting as an antioxidant but is also required as a structural component of a number of first line defence antioxidant molecules^55,56^. Studies have shown that zinc deficiency leads to increased oxidative stress *in vitro* as well as in plasma of *in vivo* animal models^28–30^. Furthermore, zinc concentrations have also been shown to be positively correlated with superoxide dismutase concentrations in cord blood^57^. Zinc deficiency in this model did not increase the amount of lipid peroxidation within the placentas which is not entirely surprising as a similar result in human FGR placentas has been documented^34,58^ and may reflect the placenta’s ability to rapidly metabolise 4HNE^59^. Furthermore, there did not appear to be a disruption to the expression of Cu/Zn-SOD in the near term placenta. However, conservation of zinc within the gestational tissues may have ameliorated any effect on Cu/Zn-SOD expression or function characterised in other studies^60,61^. There was however, a significant elevation in DNA damage, particularly within the giant trophoblast cells present within the labyrinth zone of the placentas suggesting possible disruption to DNA repair mechanisms within these cells similar to that previously seen in cell culture models of zinc deficiency^17,18^. The giant trophoblast cells within the labyrinth zone function to produce hormones such as placental lactogen II and are positioned in order to facilitate delivery of hormones and nutrients into the fetal circulation^62,63^. Consequently, increased DNA damage would likely reduce cellular integrity which could then effect aspects of giant trophoblast function and nutrient delivery, potentially amplifying already poor nutrient delivery from the maternal circulation resulting in fetal growth restriction.

There may also be a strong relationship between increased DNA damage in the placentas and the reduction of both placental and fetal iron in the zinc-deficient dams. Iron deficiency results in impaired mitochondrial function and increased oxidative stress in a number of different tissues^31,32^, hence it is reasonable to assume a reduction in placental iron is influencing oxidative stress pathways in a similar manner. Perturbed iron transport in the placentas also suggested a shift in iron transport out of the placenta occurring as part of a response to conserve zinc. Similar to our study, placental Tfrc expression has been shown to be inversely related to fetal liver iron concentrations^64^ suggesting a dialogue between the fetus and placenta which mediates iron transport. However, how this may be mediating zinc conservation within the gestational tissues - so why iron is reduced is unknown. Iron is known to be extremely important in supporting cell growth and metabolism^65,66^ and therefore reduced transport to the fetus would contribute to growth reduction. Previous studies have shown an accumulation of iron in tissues including the placenta and fetus in zinc deficient animal models^67,68^. The disparity with the current results likely reflects that previous studies utilised models of severe zinc restriction (˂0.5 mg/kg). Nevertheless, this new data further highlights a strong association between zinc and iron transport in the placenta worthy of further investigation.

In conclusion, this study describes the effects of moderate maternal zinc deficiency on placental development and function, as well as responses of the maternal cardiovascular system to reduced zinc. Furthermore, the work implicates mechanistic pathways involving altered kidney and immune function by which inadequate zinc intake and stores during pregnancy impact on offspring phenotype. Zinc deficiency contributes substantially to the predicted 3.5 million deaths per year that are attributed to child and maternal undernutrition^69^. Reducing the prevalence of zinc deficiency in developing countries was a key aspect of the Millennium Development Goal 1^70^. Our findings highlight the importance of adequate zinc status in pregnancy as deficiency impairs fetal growth. Since the trajectory of placental development is set in very early pregnancy, our data point to a need for further research to define the most critical window for zinc effects in pregnancy, and to develop strategies to reduce the impact of zinc deficiency in vulnerable women.

## Material and Methods

### Animals and diets

Seven-week-old C57Bl/6 J female mice (Laboratory Animal Services, University of Adelaide) were fed either a zinc replete diet containing 40 mg/kg zinc (Modified AIN93G, SF09-093; Specialty Feeds) or a marginally zinc deficient diet containing 10 mg/kg zinc (Zinc modified AIN93G, SF14-087; Specialty Feeds) as determined by inductively coupled plasma atomic emission spectrometry (ICP-MS). Animals were housed and maintained at Laboratory Animal Services, University of Adelaide on a 12 h: 12 h light-dark cycle with both water and food provided *ad libitum*. After six weeks on the respective diets, females were randomly allocated to either blood pressure monitoring by radio-telemetry (Cohort 1; n = 8) or analysis of placental and fetal growth (Cohort 2; n = 30). Mice in Cohort 2 were mated with a C57BL/6 J male fed standard chow (Specialty Feeds). The presence of a vaginal copulatory plug was designated gestational day 0.5 (GD0.5). Given the difficulty associated with implanting the radio-telemetry probe into very small mice, the mice in Cohort 1 were maintained on respective diets until 21 g (17-18 weeks-old), when radio-telemetry surgery was performed. All animals were maintained on diets until conclusion of experiments. Animal use complied with the Australian Code of Practice for the Care and Use of Animals and ethics approval was obtained from the University of Adelaide Ethics Committee (Approval #M-2014-84).

### Elemental Analysis

Elemental analysis on maternal plasma, placental and fetal tissues was undertaken to determine the effect of dietary zinc restriction on calcium, potassium, magnesium, sodium, sulphur and phosphorus, determined using inductively-coupled plasma optical emission spectrometry (ICP-OES) (Agilent 5100 ICP-OES; *CSIRO Analytical Services, South Australia*) and zinc, iron, copper, and selenium determined by ICP-MS (Agilent 7700 ICP-MS). Details of the sample preparation procedure are provided in *Supplementary Materials and Methods*. Disruptions to long term zinc stores were analysed by measuring liver metallothionein at GD18.5 and 21 days post-birth using a ^109^Cd/heme affinity assay^71^.

### Placental and Fetal Characteristics

Post-mortem was performed on the dams in cohort 2 between 1000–1200 h on GD18.5, described in full in *Supplementary Materials and Methods*. Maternal, placental and fetal tissue was collected and the number of total, viable and resorbing implantation sites was counted. From each dam, 2 placentas were fixed and 5 µm thickness full-face sections were stained with Masson’s Trichrome following standard protocols in order to determine the mid sagittal total, labyrinth zone and junctional zone cross sectional areas. In depth analysis of labyrinth structure was performed using immunohistochemistry (IHC) as previously described^72,73^ and details of antibodies and dilutions used are provided in Supplementary Table S5. Microarray, qPCR, IHC and Western blot analysis were employed to analyse gene and protein parameters and are detailed in *Supplementary Materials and Methods*. Microarray data have been deposited to NCBI GEO under the accession GSE97112.

### Radio-telemetry surgery, blood pressure monitoring and kidney analysis

Dams in cohort 1 were anaesthetised (2.5% isofluorane and 1 L/minute O_2_, then maintained at 1.5% isofluorane and ½ L/minute O_2_; Attane^TM^ Isoflurane, *Bomac*) and a radio-telemetry probe (PAC10, *Data Sciences International*) was inserted into the left carotid artery. The probe body was placed subcutaneously on the right flank. Mice recovered for 10 days, then MAP and HR were monitored for 10 seconds every 10 minutes for 5 days prior to the dams being placed with males. The presence of a copulatory plug designated GD0.5 and MAP and HR continued to be measured through pregnancy, birth and lactation. Pups were counted on the day of birth and weighed at 3, 5 and 7 days post-birth and at weaning. 21 days post-birth, dams and pups were killed and post-mortems performed between 1000–1200 h to collect maternal and pup tissues as described in *Supplementary Materials and Methods*.

To further investigate the effects of zinc deficiency on maternal blood pressure, the left kidney was fixed as per the protocol outlined in *Supplementary Materials and Methods*. The right kidney was snap-frozen for molecular analyses. 5 µm full-faced, mid-sagittal sections were stained using Masson’s Trichrome in order to determine the cortex and medullary zone area. Picrosirius red staining was used to determine collagen deposition and Jones’ membrane staining was used to assess glomeruli morphology. In order to understand effects on aspects of renal function, 91 genes known to be associated with blood pressure regulation were analysed using qPCR. The staining procedures as well as details of qPCR gene expression analysis, IHC and Western blot analysis of protein expression are detailed in *Supplementary Materials and Methods*.

### Statistics

All statistical analysis was performed in R (v3.1.1)^74^ using Mann-Whitney Test unless otherwise specified. Maternal organ weights, fetal and placental weight were corrected for viable litter size using the *lme* function in the *nlme* package to generate predicted values based on an average litter size of 7, then Mann-Whitney test was used to calculate exact *P*-values based on the predicted results. Significant differences in 24 h MAP and HR between the two diet groups were determined using a generalized additive mixed-model, as detailed in *Supplementary Materials and Methods*. Results are reported as median and interquartile range unless otherwise stated.

### Data Availability

Microarray data have been deposited to NCBI GEO under the accession GSE97112.

## Electronic supplementary material

Supplementary Information
