# Supplementary material for: Zinc is a critical regulator of placental morphogenesis and maternal hemodynamics during pregnancy in mice
Source: Sci Rep. 2017 Nov 9;7:15137. doi: 10.1038/s41598-017-15085-2 (PMC5680205; doi:10.1038/s41598-017-15085-2)
Supplement: Supplementary file 1 — Supplementary Information [file 41598_2017_15085_MOESM1_ESM.docx]

**Supplementary Information**

**Title -** Zinc is a critical regulator of placental morphogenesis and maternal hemodynamics during pregnancy in mice

**Authors -** Rebecca L Wilson, Shalem Y Leemaqz, Zona Goh, Dale McAninch, Tanja Jankovic-Karasoulos, Gabriela E Leghi, Jessica A Phillips, Katrina Mirabito Colafella, Cuong Tran, Sean O’Leary, Sam Buckberry, Stephen Pederson, Sarah A Robertson, Tina Bianco-Miotto, Claire T Roberts

**Supplementary Figures and Tables**


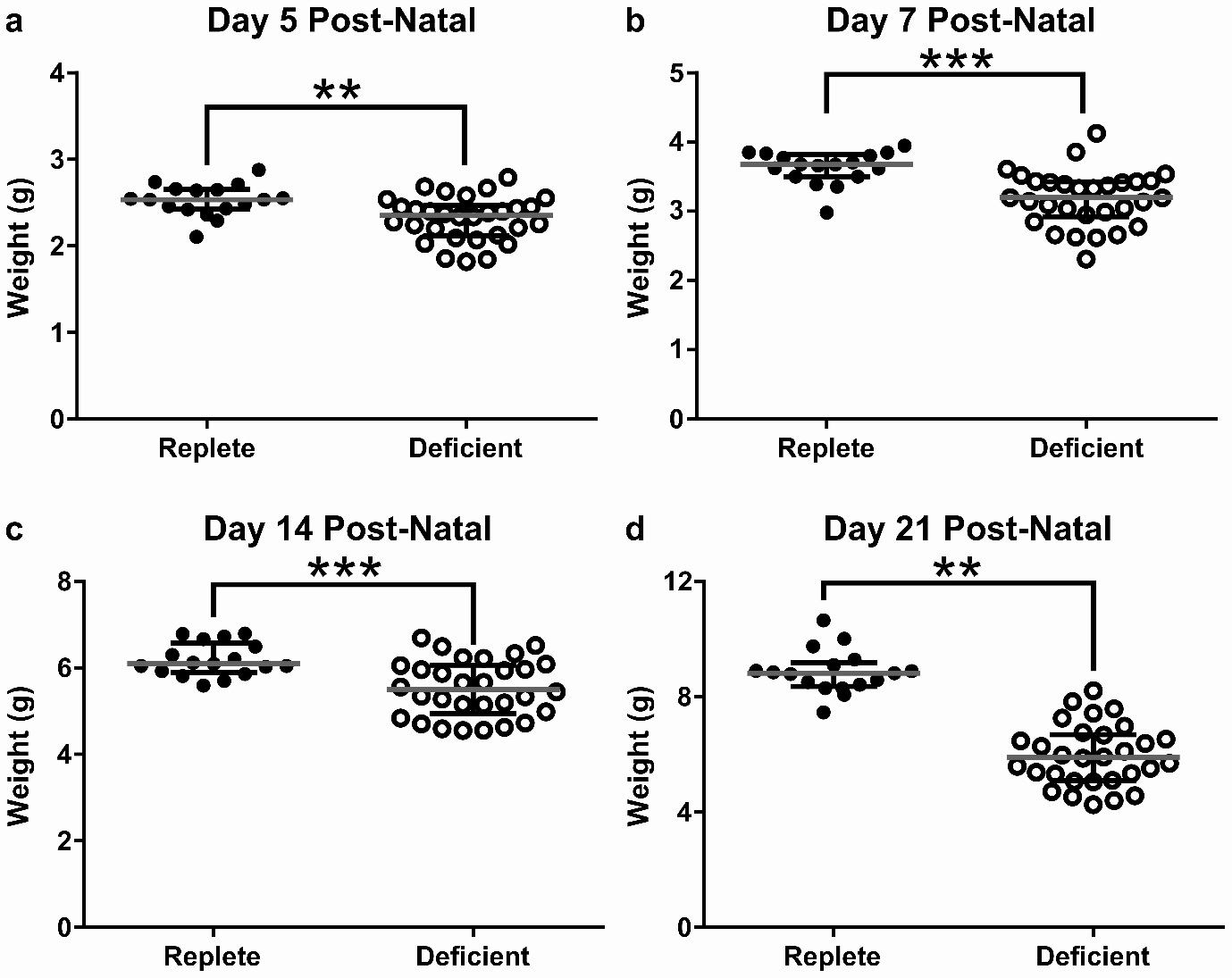


Figure S1. Maternal zinc deficiency reduced pup weight gain during lactation. Pup weight was significantly lighter in those from the zinc-deficient dams at 3 day post-natal (a), 1 week post-natal (b), 2 weeks post-natal (c) and at weaning, 3 weeks post-natal (d). Data are median and interquartile range (n = 17 and 30 pups from 4 zinc-replete and 6 zinc-deficient dams, respectively). Statistical significance was determined using Mann-Whitney Test on data based on an average litter size of 7.00. ***P*˂0.01, ****P*˂0.001.


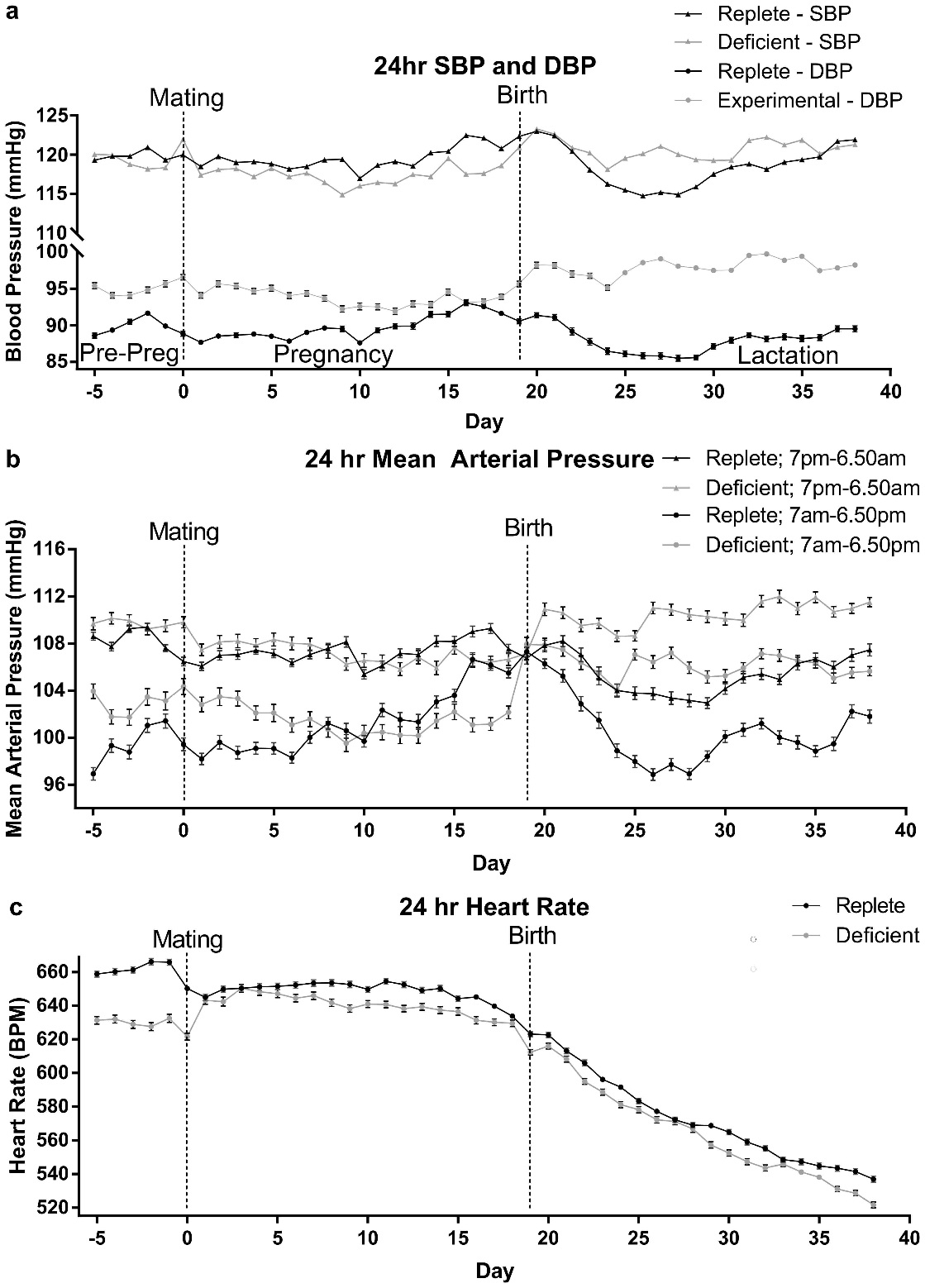


Figure S2. The effect of zinc on maternal systolic blood pressure (SBP) and diastolic blood pressure (DBP) as well as the influence of the day-night cycle. The SBP and DBP profiles prior to pregnancy, during pregnancy and in lactation were abhorrent within the animals fed the zinc deficient diet compared to those on a zinc replete diet (a). A clear circadian rhythm was observed in both diet groups as evident by higher 24 h mean arterial pressure (MAP) between the hours of 7pm to 6.50am compared to 7am to 6.50pm (b). During pregnancy differences in MAP between the two diet groups were driven by significant differences in MAP between the day light hours (7am-6.50pm). No significant differences between MAP in the night hours (7pm-6.50am) were observed between the two diet groups across pregnancy. 24 h heart rate (HR) was decreased in the zinc-deficient dams across all time periods assessed (c). Each data point represents the average 24 h SBP/DBP (a), MAP (b) or average 24 h HR (c) for each diet group ± SEM. n = 4 zin-replete and 4 zinc-deficient dams. Statistical differences were determined using a general additive model.


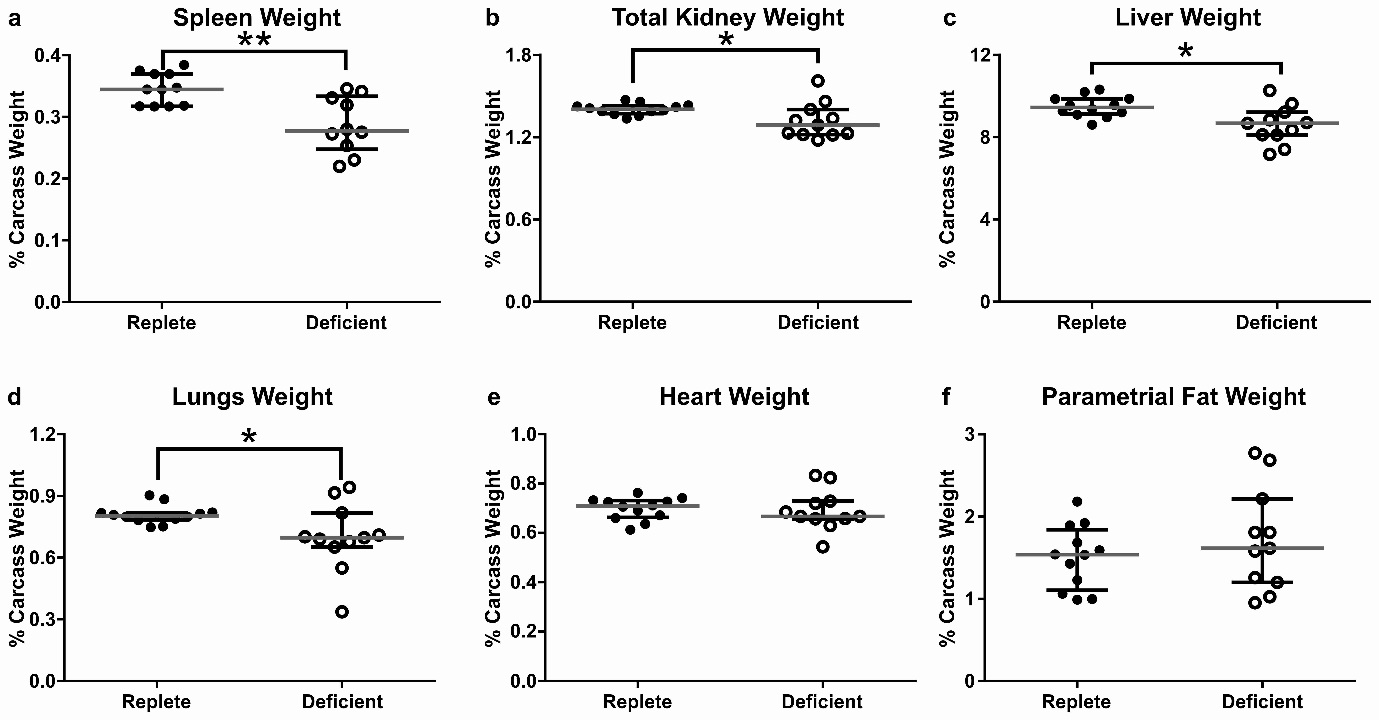
Figure S3. Liver (a), total kidney (b), spleen (c) and lungs (d) weight as a percentage of carcass weight were all reduced in the zinc-deficient dams at GD18.5. Heart (e) and parametrial fat (f) weight as a percentage of carcass weight did not change between the two diet groups. Data are median and interquartile range (n = 12 zinc-replete and 11 zinc-deficient dams). Statistical significance was determined using Mann-Whitney Test on data based on an average litter size of 7.00. **P*˂0.05, ***P*˂0.01.


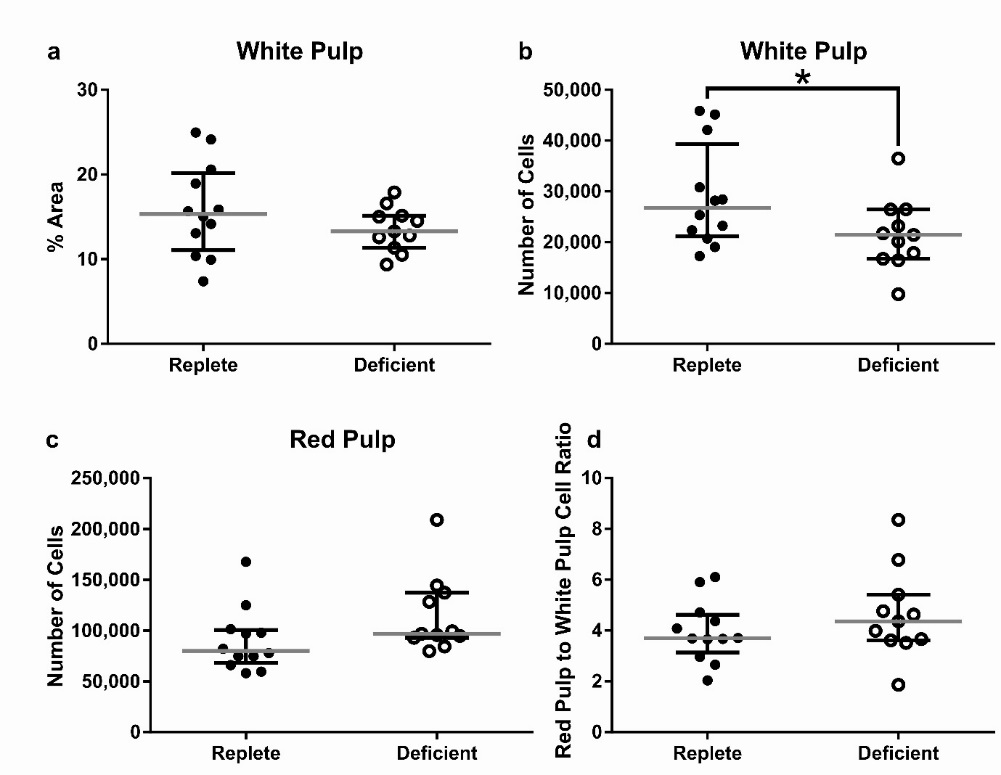


Figure S4. Histological analysis was used to determine the effects of zinc deficiency on spleen morphology at GD18.5. There was no difference in the percentage area of white pulp within the spleens between the two diet groups (a) but there was a decrease in the number of cell present within the white pulp of the zinc-deficient animals when compared to the zinc-replete animals (b). Analysis of the red pulp revealed no difference in the number of cells present within this area of the spleen (c) nor was there any difference in the red pulp to white pulp cell ratio (d). Data are median and interquartile range (n = 13 zinc-replete and 12 zinc-deficient dams). Statistical significance was determined using Mann-Whitney Test. **P*=0.051.


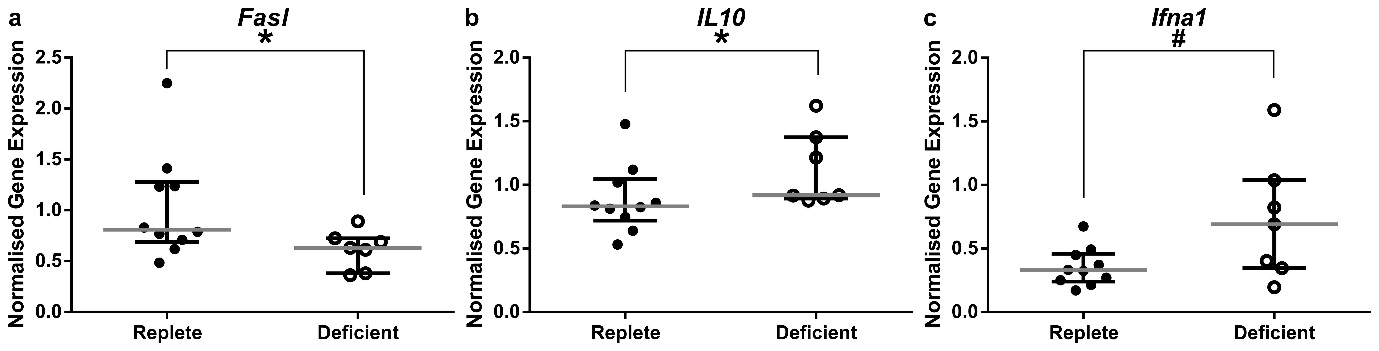


Figure S5. Maternal zinc deficiency resulted in differential gene expression of a number of cytokines specific for certain T cell populations in the spleens at GD18.5. A decrease in *Fas ligand* (*Fasl*) (a) and an increase in *interleukin 10* (*IL10*) expression (b) was observed in the spleens of the zinc-deficient dams. There was also a trend for an increase in the expression of *interferon alpha 1* (*Ifna1*) (c). Data are median and interquartile range (n = 10 zinc-replete and 7 zinc-deficient dams). Statistical significance was determined using Mann-Whitney Test. **P*˂0.05, #*P* = 0.07.

**
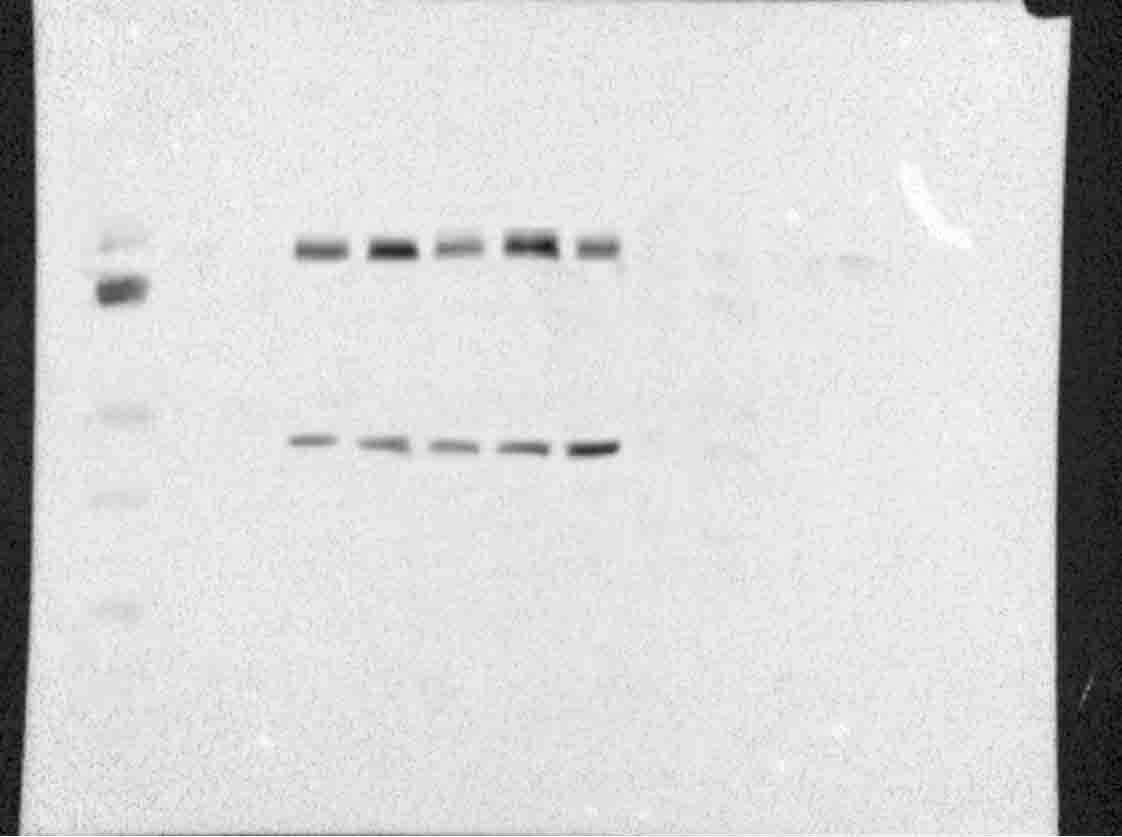
**

Figure S6. Full length western blot image of placental tfrc expression. Tfrc protein band was observed at ~100kD whilst the corresponding β-actin band was observed at ~48kD.

**Table S1.** Differences in elemental compositions of maternal plasma, placental tissue and fetal tissue

| Element | Plasma Content | Placental Content | Fetal Content |
| --- | --- | --- | --- |
| Iron  (mg/L; mg/kg) | 520.02 [493.4-787.1] vs. 457.81 [357.6-635.3] | 63.20 [54.6-85.4] vs. 49.60 [45.1-66]* | 35.88 [35-39.4] vs. 28.94 [25.6-35]** |
| Potassium  (mg/L; mg/kg) | 32.28 [29.7-40.1] vs. 41.90 [34.4-51.6]* | 2287.46 [1683.7-2932.7] vs. 2109.85 [1860.1-2685.1]^NS^ | 2345.94 [2171.6-2511.4] vs. 2421.07 [2259.4-2494.2]^NS^ |
| Sulphur  (mg/L; mg/kg) | 132.12 [120-139.8] vs. 149.90 [140-164]** | 1422.56 [910.4-1724] vs. 1299.33 [1109.1-1570.6]^NS^ | 1146.06 [1108.7-1190.9] vs. 1139.88 [1123-1160.1]^NS^ |
| Phosphorus  (mg/L; mg/kg) | 16.42 [14.9-18] vs. 19.61 [18.6-22.4]** | 2148.78 [1655.7-2406.1] vs. 2134.85 [1684.9-3147.5]^NS^ | 2552.25 [2336.8-2684.8] vs. 2658.39 [2571-2720.6]^NS^ |
| Copper  (µg/L; µg/kg) | 238.29 [207.9-252] vs. 281.02 [234.8-296.2]* | 2575.66 [1766.3-3023.9] vs. 3024.66 [1874.7-3734.8]^NS^ | 2233.05 [2022.5-2387] vs. 2184.70 [2017.6-2484.3]^NS^ |
| Sodium  (mg/L; mg/kg) | 629.53 [606.5-650] vs. 654.81 [641.1-680.1]^NS^ | 1118.93 [902.3-1751] vs. 1149.60 [759.8-1449.3]^NS^ | 1694.22 [1647.8-1739.6] vs. 1768.22 [1703.1-1808.6]* |
| Data are presented as median [interquartile range]. n = 10 zinc-replete and 7 zinc-deficient dams and n= 19 and 13 placentas and fetuses from zinc-replete and zinc-deficient dams, respectively. Statistical significance was determined using a Mann-Whitney test. **P*˂0.0**5,** ***P*˂0.0**1** NS, not significant. | | | |

**Table S2.** Elemental composition of the zinc-replete and zinc-deficient diets as determined by mass spectrometry

| Element | Zinc-replete Diet  (40 mg/kg zinc) | Deficient Diet  (10 mg/kg zinc) | *P* value |
| --- | --- | --- | --- |
| Iron (mg/kg) | 41 ± 7.3 | 35 ± 3.3 | NS |
| Manganese (mg/kg) | 10 ± 1.7 | 17 ± 13.7 | NS |
| Copper (mg/kg) | 8 ± 0.2 | 5 ± 1.2 | *P*˂0.05 |
| Zinc (mg/kg) | 39 ± 0.3 | 11 ± 1.3 | *P*˂0.001 |
| Calcium (mg/kg) | 6500 ± 141.4 | 6133 ± 115.5 | NS |
| Magnesium (mg/kg) | 715 ± 7.1 | 660 ± 26.5 | NS |
| Sodium (mg/kg) | 1850 ± 14.1 | 1963 ± 63.5 | NS |
| Potassium (mg/kg) | 4700 ±141.4 | 7267 ± 321.5 | *P*˂0.01 |
| Phosphorus (mg/kg) | 2300 ± 141.4 | 3600 ± 200.0 | *P*˂0.01 |
| Sulphur (mg/kg) | 3400 ± 0 | 3733 ± 152.8 | NS |
| Aluminium (mg/kg) | 4 ± 0.9 | 7 ± 0.2 | NS |
| Elemental analysis of the diet composition was performed on two independent samples of zinc-replete chow and three independent samples of zinc-deficient chow and analysed by inductively-coupled plasma atomic emission spectrometry. Data are mean ± standard deviation. Statistical significance was determined using a two-tailed T-test, assuming unequal variances. NS, not significant | | | |

**Table S3.** Genes differentially expressed between zinc-replete and zinc deficient placentas

| Gene (Gene symbol) | Fold change | Deficient Expression | *P* value | FDR |
| --- | --- | --- | --- | --- |
| *Transferrin Receptor* (*Tfrc*) | 8.59 | **↑** | 8.77E-06 | 0.112 |
| *Sclerostin domain containing 1* (*Sostdc1*) | 2.70 | **↑** | 4.10E-05 | 0.182 |
| *Elongation of very long chain fatty acids-like 4* (*Elovl4*) | 2.57 | **↑** | 4.70E-05 | 0.182 |
| *Interferon-related developmental regulator 1* (*Ifrd1*) | 1.69 | **↓** | 6.95E-05 | 0.182 |
| *RIKEN cDNA 1700023L04* (*1700023L04Rik*) | 2.40 | **↓** | 7.13E-05 | 0.182 |
| *WD repeat domain 60* (*Wdr60*) | 1.67 | **↓** | 1.09E-04 | 0.231 |
| *TSC22 domain family, member 3* (*Tsc22d3*) | 1.74 | **↓** | 1.27E-04 | 0.232 |
| *Glutamate receptor interacting protein 1* (*Grip1*) | 2.21 | **↓** | 1.67E-04 | 0.266 |
| *Catsper channel auxiliary subunit delta* (*Catsperd*) | 2.80 | **↓** | 2.29E-04 | 0.324 |

**Table S4.** Effect of zinc deficiency on renal expression of seven genes at GD18.5 as quantified by qPCR

| Gene | Normalised Expression (zinc-replete vs. zinc-deficient) | Difference in Deficient | *P* value |
| --- | --- | --- | --- |
| *Ace* | 1.15 [1.04-1.75] vs. 0.97 [0.65-1.30] | ↓ 16% | 0.031 |
| *Actg2* | 0.26 [0.14-0.52] vs. 0.68 [0.43-1.08] | ↑ 162% | 0.011 |
| *Aldh3a2* | 1.36 [1.18-1.50] vs. 1.11 [1.01-1.32] | ↓ 18% | 0.031 |
| *Braf* | 1.46 [1.28-1.74] vs. 1.07 [1.03-1.31] | ↓ 27% | 0.005 |
| *Calcoco1* | 1.57 [1.26-1.71] vs. 1.01 [0.85-1.09] | ↓ 36% | 0.002 |
| *Cbx7* | 1.45 [1.28-1.74] vs. 1.03 [0.76-1.17] | ↓ 29% | 0.003 |
| *Itpr2* | 1.03 [0.77-1.37] vs. 0.58 [0.55-1.03] | ↓ 44% | 0.05 |
| Data represented as median [IQR]. Gene expression was normalised to *Tbp* and *Gapdh*. n = 9 and 7 kidneys from zinc-replete and zinc-deficient dams, respectively. Statistical significance was determined using a Mann-Whitney test. | | | |

**Table S5**. Antibodies and dilutions used for immunohistochemistry and western blots

| **Antigen** | **Supplier** | **Species** | **Clone** | **IHC Dilution** | **Antigen Retrieval** | **Western Blot Dilution** |
| --- | --- | --- | --- | --- | --- | --- |
| Vimentin | Dako | Monoclonal Mouse | Vim 3B4 | 1/10 | Yes, Pronase |  |
| Pan-cytokeratin | Chemicon | Monoclonal Mouse | AE1/AE3 | 1/100 | Yes, Pronase |  |
| ACE | Santa Cruz | Polyclonal Rabbit | H-170 | 1/50 | Yes, Citrate | 1/250 |
| Tfrc | Thermo Fisher | Monoclonal Mouse | H68.4 | 1/500 | Yes, Pronase | 1/1500 |
| 4-HNE | Alpha Diagnostics | Polyclonal Rabbit |  | 1/500 | Yes, 0.1% Triton & 0.1% Tween20 in TBS | 1/1000 |
| 8-OHdG | JaICa | Monoclonal Mouse | N45.1 | 1/100 | Yes, Citrate |  |
| Cu/Zn-SOD | Sigma | Polyclonal Rabbit | SOD1 | 1/2000 | Yes, Citrate |  |
| HSP-90 | Santa Cruz | Polyclonal Rabbit | H-114 |  |  | 1/2000 |
| β-actin | Santa Cruz | Polyclonal Rabbit |  |  |  | 1/1000 |

**Supplementary Materials and Methods**

**Post-mortems and pregnancy parameters**

Dams were anaesthetised with an intraperitoneal injection of 0.75 µg/g Avertin (2,2,2-tribromomethanol, *Sigma Aldrich*) in 2-methyl-2-butanol. Blood was collected in a heparinised tube via retro-orbital bleed to which plasma was extracted after centrifugation at 10,000 x g and stored at -80°C. Dams were then killed via cervical dislocation. Placental and fetal tissue was dissected from the uterus, membranes removed and snap frozen for molecular analysis. Two placentas from each dam were randomly selected to be fixed in 4% (wt/vol) paraformaldehyde/2.5% polyvidone-40 (wt/vol) in 70 mM phosphate buffer [pH 7.0] overnight at 4°C, washed in 4 changes of PBS and paraffin-embedded. Maternal tissues including spleen, liver, kidneys, lungs, heart and parametrial fat were also excised with the left kidney and heart being fixed following the same procedure as the placentas.

**Elemental analysis of maternal plasma and placental and fetal tissues**

Briefly, 200 μL of plasma, approximately 25 mg of placental tissue and the torso of the GD18.5 fetus were digested in Teflon vessels at room temperature in concentrated nitric acid (~70% HNO_3_) for approximately 2 h before further digestion under pressure at approximately 90°C overnight. The following day, the heat was further increased to approximately 140°C and left a further 24 h. The samples were then diluted with milliQ water and made up to 20 mL to be injected onto the relevant apparatuses for analysis. For ICP-MS, samples were run alongside two internal standards: iridium and rhodium (*Choice Analytical*) at a concentration of 200 ppb and an 8-point calibration, including blank, was carried out between 0.01 µg/L and 100 µg/L. An 8-point calibration (including blank) was also carried out between 500 mg/L and 1000 mg/L for the ICP-OES analysis.

**Kidney and spleen morphological analysis**

For kidneys, full-face mid-sagittal sections were stained using Masson’s Trichrome in order to determine the cortex and medullary zones following standard protocols. Collagen deposition was measured using picrosirius red staining. De-waxed sections were immersed in a solution of 0.1% Direct Red (*Sigma-Aldrich*) and 1.2% picric acid (*Sigma-Aldrich*) in water for 1 h, rinsed in running tap water, counter stained with Wiegert’s haematoxylin for 20 mins then dehydrated, cleared and mounted. Slides were scanned using the Nanozoomer 2.0-HT C9600-13 and random systematic sampling of the cortex area was used to select 10 images of each section at 20x magnification using the NDP View2 software (*Hamamatsu*). ImageJ Version 1.48 software (*National Institutes of Health*) colour deconvolution plugin was used to measure percentage of collagen. Glomeruli morphology was assessed using Jones Basement Membrane (PAS-M) staining kit (cat. # AR18011-2, *Dako*) following manufacturers protocol. Glomeruli basement membrane thickness, area, perimeter and roundness ((perimeter^2)/(4*π*area)) were calculated using the NDP View2 software.

For spleens, frozen sections were fixed for 10 mins in cold methanol, washed in PBS and then stained using hematoxylin and eosin following standard protocols. Slides were scanned using the Nanozoomer 2.0-HT C9600-13. Using the NDP View2 software, total spleen area and the areas of red and white pulp were measured. Random systematic sampling was used to select 10 images of each section at 80x magnification. The total number of cells in each image was counted using the Particle Analysis tool in the Image J Version 1.48 software.

**Placental and Kidney protein expression analysis**

Immunohistochemistry and western blots were used to quantitate protein expression of angiotensin converting enzyme (Ace), transferrin receptor, hydroxynonenal (4-HNE), Cu/Zn-Superoxide Dismutase (SOD) and 8-hydroxy-2’-deoxyguanosine (8-OHdG). Antibodies and dilutions are provided in supplementary table 1 (Table S5).

As for placental and kidney morphometric analyses, cut sections were de-waxed, rehydrated according to standard protocols. Where required, antigen retrieval was performed and endogenous peroxidase activity was suppressed by incubating the slides in 3% hydrogen peroxide. Primary antibodies were diluted in 5% serum in PBS and applied overnight at 4°C in a humidified chamber. Negative controls were also included by omitting the primary antibody from the diluent. Secondary antibodies were diluted in 5% serum and slides were incubated for 1 h at room temperature. Primary antibody binding was amplified using streptavadin horseradish peroxidase (strep-HRP) (*Dako*) and detected using DAB (*Sigma-Aldrich*). Video Image Analysis (VIA) software (*Leading Edge Software*) was used to calculated intensity and percent positivity of DAB staining.

For western blotting, placental and kidney tissue were homogenised in ice-cold RIPA buffer. Protein concentrations were determined using a Bradford Assay (*Bio-rad*). 25 µg of protein was run on a 10% mini PROTEAN^TM^ TGX^TM^ pre-cast gel (*Bio-Rad)* following manufacturer’s protocols and then transferred onto nitrocellulose membranes using the Criterion^TM^ Blotter (*Bio-Rad*). Membranes were incubated overnight a 4°C in a blocking solution containing 3% skim milk in TBS-T (Tris-Buffered Saline containing Tween 20) before being incubated in the relevant primary antibody for 1 h at room temperature. After thorough washing in TBS-T, membranes were further incubated with a secondary antibody conjugated with HRP for 1 h at room temperature. Protein bands were visualised by chemiluminescence with ECL (*GE Healthcare*) on the Chemidoc MP (*Bio-Rad*). Levels of protein expression were normalised to HSP-90 or β-actin.

**RNA extraction, placental microarray analysis and placenta, spleen and kidney qPCR**

Placental, spleen and kidney tissues were homogenised using a Powerlyzer with ceramic 1.4 mm beads (Mo Bio Laboratories, Inc). Total RNA was extracted using Trizol (Invitrogen) following the manufacturer’s instructions and RNA integrity was determined using the Experion (*Bio-Rad*) system.

For placental microarray, 31 placentas from 10 zinc-repletezinc-replete and 7 zinc-deficient dams were analysed as described by (56). Affymetrix Mouse Gene 2.1 ST array data were checked for quality, RMA background-corrected and quantile normalised in R (58), using the *aroma.affymetrix* package (59) and v19.0.0 of an Ensembl-derived CDF (http://brainarray.mbni.med.umich.edu/Brainarray/). Detection above background (DABG) was performed by estimating non-specific binding (NSB) properties of the antigenomic probes using a modified version of the MAT background model (60) and obtaining empirical distributions of residuals across 10 bins of fitted values. After training the model on the set of antigenomic probes, fitted NSB estimates were obtained for the PM probes based on their sequence content. Fitted NSB estimates were subtracted from the observed PM intensities on the log2 scale and residuals were compared to the empirical distributions obtained from the antigenomic probes to provide *p*-values for detection of signal above the background. Probe-level *P* values were obtained using Fisher’s method across the set of arrays, and only PM probes with a *p*-value < 0.05 were considered as containing “true” signal, giving 51.4% of probes which were able to be used for estimation of expression levels. Differential expression between diet groups was assessed using the package *limma* (61), and all *p*-values were corrected for multiple testing using the Benjamini-Hochberg method (62) to provide estimates of the false discovery rate (FDR).

The outcome of the microarray analysis was validated using qPCR on RNA samples from all placentas collected at post-mortems (n=72, 50 from zinc-replete and 32 from zinc-deficient dams). PrimerPCR^TM^ assays (Bio-Rad) specific for mouse *Transferrin Receptor* (*Tfrc*) (cat. # QMMUCID0039655) and *Transferrin* (*Trf*) (cat. # QMMUCID0061477) were purchased along with housekeeping assays for *Tbp* (cat. # QMMUCID0040542) and *Hmbs* (cat # QMMUCID0022816). *Tfrc* and *Trf* gene expression was also quantified in kidney samples from dams collected at GD18.5 (n=23, 12 zinc-replete and 11 zinc-deficient). Extracted RNA was DNase treated using TURBO DNA-free^TM^ (*Ambion*) as per manufacturer’s instructions and quality zinc-replete for DNase-treatment was double-checked using PCR for primers specific for mouse genomic DNA (56). 500 ng of DNase-treated RNA was reverse transcribed using iScript^TM^ Reverse Transcriptase Kit (*Bio-Rad*) following manufacturer’s instructions. Each cDNA sample was diluted 1:5 and added to a PCR reaction containing the relevant primer assay and Ssofast Evergreen Supermix (*Bio-Rad*). qPCR was performed in triplicate following cycling conditions recommended by the manufacturer. Data was analysed using the Bio-Rad CFX Manager Software v3.1 and all gene expression data was normalised to *Tbp* and *Hbms*.

Expression of genes associated with blood pressure zinc-replete and immune function were assessed in the kidneys and spleens of dams collected at GD18.5, respectively (n=17, 10 replete and 7 deficient). RNA from each kidney or spleen was DNAse treated as previously mentioned and 4 µg of DNase-treated RNA was reverse transcribed using iScript^TM^ Advanced cDNA Synthesis Kit (*Bio*-Rad). cDNA was diluted 1:15 and added to a reaction mix containing SsoAdvanced^TM^ SYBR^®^ Green Supermix (*Bio-Rad*) and then added to PrimePCR^TM^ Pre-eclampsia Tier 1 M384 plates (cat. # 10039501, *Bio-Rad*) for kidney RNA or PrimePCR^TM^ Immune Tier 1 M384 plates (cat. # 10029798, *Bio-Rad*) for spleen RNA. Plates were run on the CFX384 PCR machine (*Bio-Rad*) with cycling conditions according to the manufacturer’s protocol. The Bio-Rad CFX Manager Software v3.1 was used to analyse the data. For kidney gene expression, data was normalised to *Tbp* and *Gapdh* while spleen gene expression was normalised to *Hprt* and *Gapdh.*

**Statistics for radio-telemetry data**

A generalized additive mixed model was fitted to the 24 h MAP, SBP, DBP, pulse pressure and HR data in order to determine the effects of the diet on blood pressure using the gamlss R package in Revolution R Open (RRO) 3.2.0. For 24 h MAP, the model was adjusted for age, weight, log of activity and heart rate using a non-linear time trend using cubic spline. A random effect was also fitted for each dam. The estimated means of the two diet groups across days of gestation were calculated and the profile for each pregnancy state (pre-pregnancy, pregnancy; including mating and birth and lactation), as well as within pregnancy based on pivotal placental development events (day 0-5, 6-10 and 11-19) were examined.
